# Lung Ultrasound Is More Sensitive for Hospitalized Consolidated Pneumonia Diagnosis Compared to CXR in Children

**DOI:** 10.3390/children8080659

**Published:** 2021-07-29

**Authors:** Ioana Mihaiela Ciuca, Mihaela Dediu, Monica Steluta Marc, Mirabela Lukic, Delia Ioana Horhat, Liviu Laurentiu Pop

**Affiliations:** 1 Pediatric Department, University of Medicine and Pharmacy “Victor Babes”, 300041 Timisoara, Romania; ciuca.ioana@umft.ro (I.M.C.); dediu.mihaela@umft.ro (M.D.); pop.liviu@umft.ro (L.L.P.); 2 Pediatric Pulmonology Unit, Clinical County Hospital, 300226 Timisoara, Romania; 3 Pulmonology Department, University of Medicine and Pharmacy “Victor Babes”, 300041 Timisoara, Romania; mariamirabela_mal@yahoo.com; 4 ENT Department, University of Medicine and Pharmacy “Victor Babes”, 300041 Timisoara, Romania; deliahorhat@yahoo.com

**Keywords:** lung ultrasound, pneumonia, children, chest X-ray, consolidation, community-acquired pneumonia

## Abstract

Background: Pneumonia is the leading cause of death among children; thus, a correct early diagnosis would be ideal. The imagistic diagnosis still uses chest X-ray (CXR), but lung ultrasound (LUS) proves to be reliable for pneumonia diagnosis. The aim of our study was to evaluate the sensitivity and specificity of LUS compared to CXR in consolidated pneumonia. Methods: Children with clinical suspicion of bacterial pneumonia were screened by LUS for pneumonia, followed by CXR. The agreement relation between LUS and CXR regarding the detection of consolidation was evaluated by Cohen’s kappa test. Results: A total of 128 patients with clinical suspicion of pneumonia were evaluated; 74 of them were confirmed by imagery and biological inflammatory markers. The highest frequency of pneumonia was in the 0–3 years age group (37.83%). Statistical estimation of the agreement between LUS and CXR in detection of the consolidation found an almost perfect agreement, with a Cohen’s kappa coefficient of K = 0.89 ± 0.04 SD, *p* = 0.000. Sensitivity of LUS was superior to CXR in detection of consolidations. Conclusion: Lung ultrasound is a reliable method for the detection of pneumonia consolidation in hospitalized children, with sensitivity and specificity superior to CXR. LUS should be used for rapid and safe evaluation of child pneumonia.

## 1. Introduction

Respiratory infections have the greatest prevalence in pediatric pathology, with pneumonia being the leading cause of death in children worldwide [1]. The worldwide death rate in children with pneumonia is significant [2], being considerably increased in developing and undeveloped countries [3,4,5]. The evolution of the disease depends on early diagnosis and the appropriate subsequent therapy. Community-acquired pneumonia (CAP) diagnosis includes—aside from clinical signs [6], which are not specific [7]—a validation of the parenchymal inflammation, commonly expressed by consolidation on standard chest X-ray (CXR) investigation. CXR was used for decades in pneumonia diagnosis, being considered the most specific investigation, even if not always correctly interpreted [8]. Although the actual guidelines do not recommend the routine use of CXR for suspected pneumonia [9,10], current practice reveals the frequent use of CXR in suspected child pneumonia [11]. Nevertheless, its associated risk of irradiation is not negligible, especially among children, and would be best avoided when possible [12,13].

Although, at first, the lung was considered to be an inappropriate organ for evaluation by ultrasonography [14], the contrary was demonstrated by several studies [5,7,10,12,15]. Today, chest ultrasound has a confirmed utility in the detection of pleurisy, with a superior efficiency compared to chest radiography, as well as pneumonia, pneumothorax, or acute distress respiratory syndrome [16,17], underlying its role in emergency medicine. Not only acute pathology benefits from LUS accuracy, but also chronic lung diseases such as pulmonary fibrosis [18,19], tuberculosis [20], or cystic fibrosis [21].

Lung ultrasound proved to be a reliable, non-irradiating, and accessible method for CAP diagnosis in adults, the point-of-care lung ultrasound being an accurate instrument that can be considered to be a significant diagnostic approach for community-acquired pneumonia [22] in children [23], as confirmed by several studies [10,12,15,16,24]. As for imagistic diagnosis of consolidations, there are studies showing the LUS has a better sensitivity in detecting smaller consolidations—especially <1 cm—compared with CXR [20,24], and some research states that larger consolidation dimensions would be suggestive of CAP [25,26].

Our study evaluated the reliability of LUS compared to CXR in consolidated pneumonia in hospitalized children.

## 2. Materials and Methods

### 2.1. Study Population

Children with clinical signs of bacterial pneumonia—consisting of fever, polypnea, tachypnea, chest pain, chest retractions, and cough—as well as symptoms and/or the presence of crackles (rales) at the auscultatory examination, in which the suspicion of pneumonia met the WHO criteria [6] (clinically defined as age-specific tachypnea and/or chest indrawing) for diagnosis of pneumonia, were included in the study. Pneumonia severity signs were defined as follows: presence of polypnea defined for age, difficulty breathing, chest retraction, and hypoxemia expressed by peripheral oxygen saturation < 92% [27]. Patients with clinical suspicion of acute asthma exacerbation, bronchiolitis, recurrent wheeze, chronic lung pathology, or immunodeficiency were excluded.

Each parent and, in cases over 12 years old, each child, signed the informed agreement on the acceptance to enter the study, in accordance with the Declaration of Helsinki, and the study was approved by the Ethics Committee of the Clinical County Hospital no 15/2017.

### 2.2. Lung Ultrasound Equipment and Methodology

Patients underwent pulmonary ultrasound using 1 of 3 probes, depending on the age of the child and the thickness of the adipose panicle: a linear probe, with a frequency of 7–12 MHz; a convex probe (3.5–5 MHz); or a 5–10 micro convex probe. We used an Alpinion E-CUBE 9 ultrasound machine. The longitudinal sections were used as ultrasound evaluation areas: right and left parasternal, mid-clavicular, and anterior and posterior axillary; in addition, scans of each intercostal space were conducted. Virtually every lung area was divided into 3 areas (upper, medial, and lower), both anterior and posterior, and the lateral areas were divided as follows: upper lateral area (axillary), medial area, and lower lateral area [10,28]. In addition, splenic and hepatic ultrasound windows were used to evaluate the costal–diaphragmatic pleural angles.

The ultrasound was performed by the pediatric pulmonologist at the time of admission, after clinical examination, before performing the chest X-ray and biochemical investigations, as well as on the 3rd–5th and 7th–10th days of evolution, being used as a method of outcome follow-up.

The presence of consolidation, viewed as “hepatization”—liver-like images or parenchymal images—with air or liquid bronchogram, anfractuous edges, and “shred” (or fractal) signs were defined as ultrasound diagnostic parameters for consolidated pneumonia [15,23,29]. The presence of bronchogram inside consolidation was considered mandatory for pneumonia in our study, in addition to the vascularization of the consolidation or perilesional B lines for a correct differentiation of atelectasis or similar pattern consolidation [30].

For pleural effusion diagnosis, the presence of hypoechoic accumulations was characteristic for simple pleural effusions, or associated with hyperechoic, inhomogeneous areas (fibrinoid detritus) in complicated pleural effusions. The presence of interstitial inflammatory syndrome was quantified by the presence of more than 3 B lines/intercostal spaces or more than one coalescent B longitudinal artifact.

Chest X-radiography, with posterior–anterior exposure, was performed after LUS, at a maximum of 12 h after ultrasound. The interpretation was completed by a radiologist blinded to the ultrasound result, according to the WHO criteria for CXR interpretation [31]. The CXR was taken on the first day of hospitalization, as a standard for the local diagnostic protocol of pneumonia, using age-appropriate radiation regimens.

In our study, only children with unfavorable evolution had CT scans performed.

### 2.3. Statistical Analysis

All data collected were analyzed using IBM SPSS Statistics 26. For the descriptive statistics, we used percentage values for qualitative variables, and means and standard deviations for quantitative variables. The Kolmogorov–Smirnov test was used to establish the non-parametric distribution of our quantitative data. Cohen’s kappa coefficient was used to evaluate the agreement relation between LUS and CXR regarding the detection of consolidation and the diagnosis of pneumonia. We interpreted the test according to Cohen’s suggestions as follows: values ≤ 0 as indicating no agreement, 0.01–0.20 as none to slight, 0.21–0.40 as fair, 0.41–0.60 as moderate, 0.61–0.80 as substantial, and 0.81–1.00 as almost perfect agreement [32]. IBM SPPS Statistic 26 also allowed us to analyze the raw data for cumulative sensitivity, specificity, negative predictive value (NPV), and positive predictive value (PPV), and 95% confidence intervals were calculated according to the efficient score method described by Robert Newcombe, based on the procedure outlined by E. B. Wilson in 1927 [33,34].

## 3. Results

### 3.1. Descriptive Data

A total of 128 patients with signs, symptoms, and pneumonia-specific clinical presentation were evaluated. From these, 54 patients were excluded (22 with viral bronchiolitis, 27 with acute asthma exacerbations and 5 with chronic lung diseases, e.g., cystic fibrosis exacerbations). Consequently, the study population was composed of 74 children (Table 1) with pneumonia. The other patients had no imagistic signs of consolidations, and as mentioned presented polypnea (57.4%) and difficulty breathing (53.7%) or decreased SpO_2_ < 95% (53.7%); 85.11% had wheezing, being further diagnosed with bronchiolitis and asthma. The clinical signs and descriptive data of patients with pneumonia and controls are shown in Table 1.

The patients were diagnosed with pneumonia without a gender-related significant predominance: 51.35% male and 48.64% female patients had pneumonia detectable by imaging. The majority of children had polypnea (87.8%) and difficulty breathing (81.08%), and more than half (58.1%) had hypoxemia at admission. Similar percentages of clinical signs were encountered among children without pneumonia.

With regard to the age distribution, the mean age of patients was 4.93 ± 3.9 years, and the highest frequency of pneumonia was in the 0–3 years age group (37.8%), closely followed in descending order by the 3–6 years group with a frequency of 33.7%, and 6–12 years with 18.9%; fewer teenagers were diagnosed with pneumonia, at a rate of 9.4% in our study.

Severe pneumonia was diagnosed in 37.8% of admitted children (Table 2).

Patients had consistent biological characteristics with bacterial inflammation, suggested by accelerated erythrocyte sedimentation rates (ESRs)—mean ESR = 41.4 ± 22.21 mm/h—and increased C-reactive protein (127.04 ± 91.62 mg%, based on a normal value of 1 mg%). The mean leukocyte level was 18.04 ± 5.51 × 10^3^/mm^3^ (Table 2).

**Figure 1:**
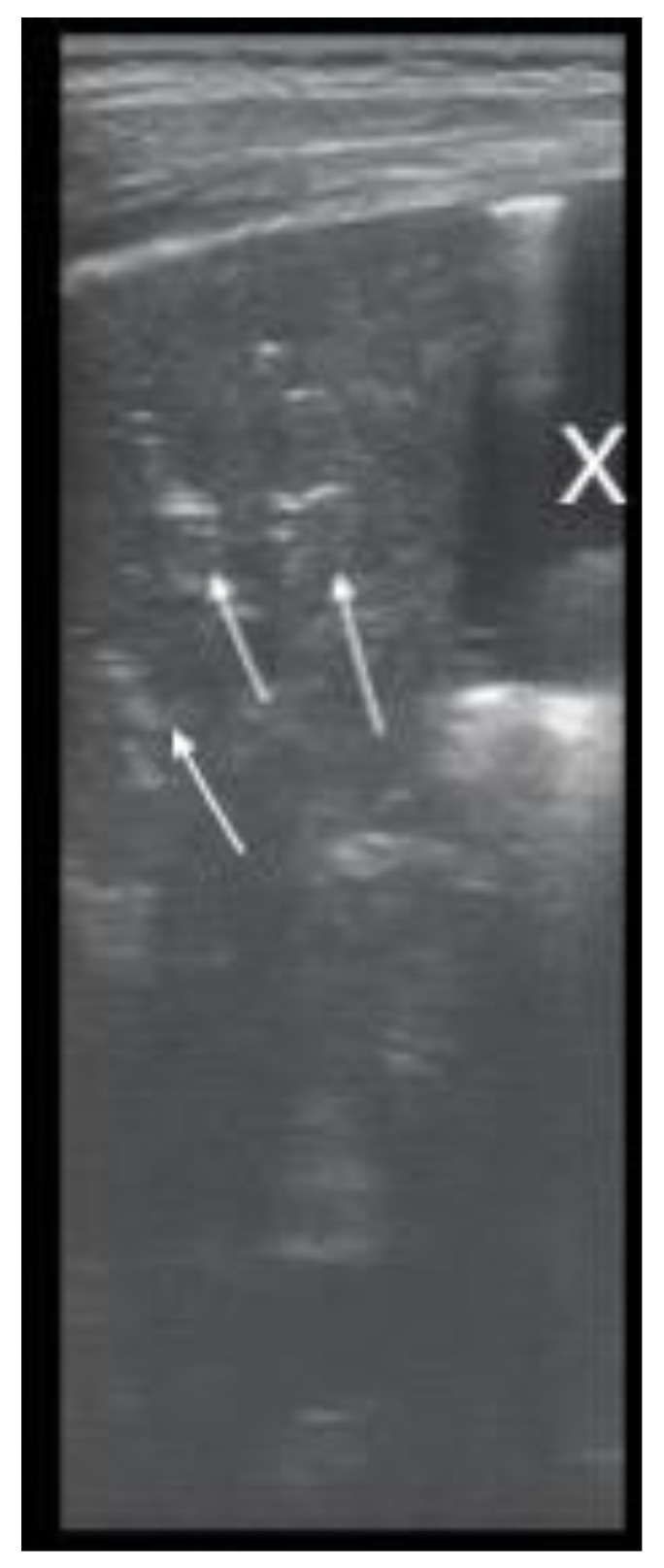
“Liver-like” Consolidation, with hyperechoic lenticular air (white arrows), consistent with air bronchogram; adjacent anechoic pleural effusion marked with X; LUS, 7–12 MHz.

**Figure 2:**
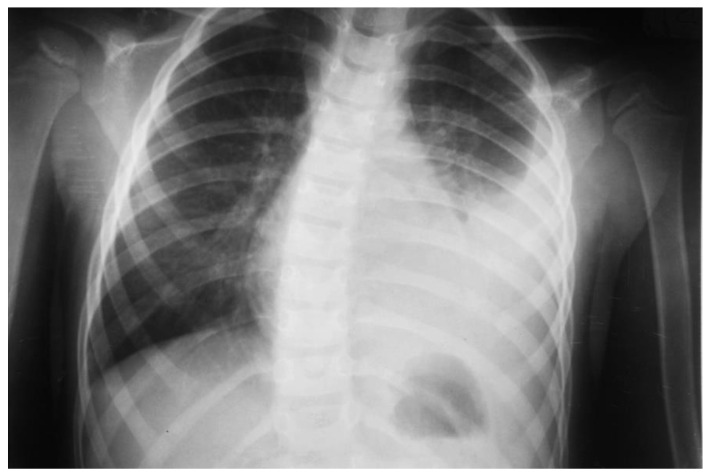
Left lower lobe pneumonia on CXR of the same case; no clear sign of pleurisy; apparently clear costal–diaphragmatic left angle.

**Figure 3:**
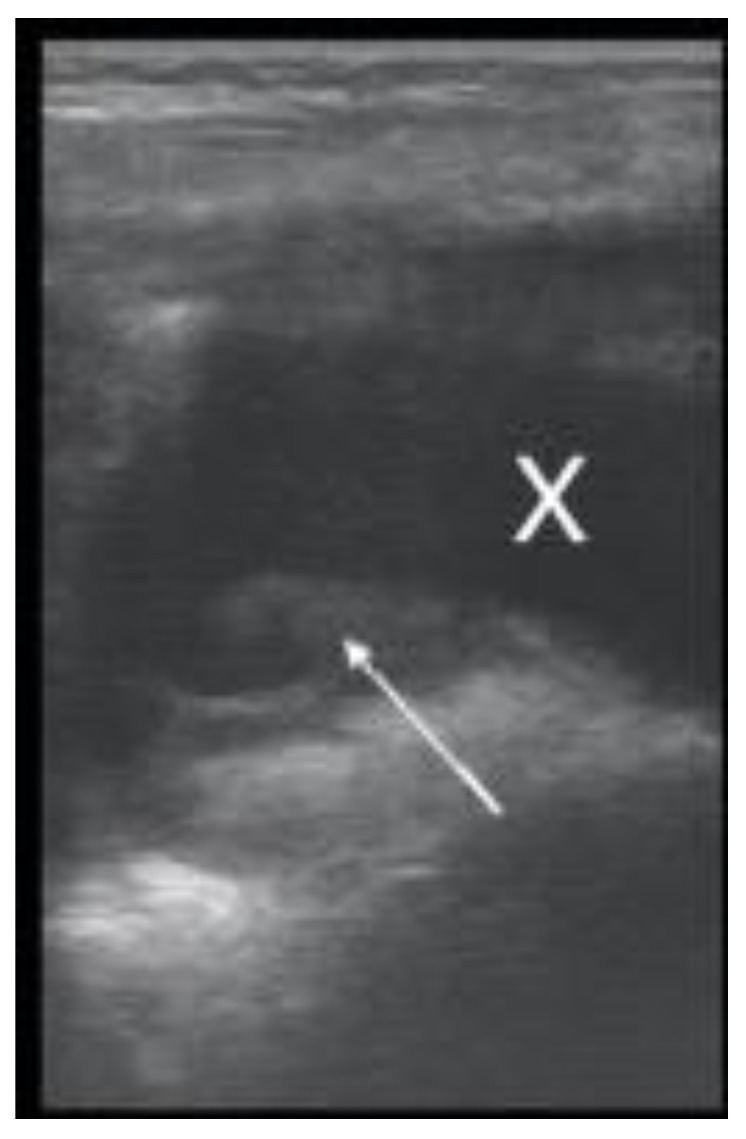
Pleural effusion with transonic liquid (marked with X) and hyperechoic plankton sign of exudative pleurisy (white arrow).

Pulmonary ultrasound detected 74 patients (100%) with consolidation and air bronchogram (Figure 1), and CXR was positive in 67 patients (Figure 2). In some patients, adjacent pleural effusion was clearly detected by LUS (Figure 3).

The presence of the air bronchogram was noticed from hyperechoic linear images, following the bronchial arborization inside the typical aspect presented in Figure 1 as hepatization, secondary to parenchymal inflammation, erasing the typical normal presence of A lines. Parapneumonic pleural effusions were sensibly detected by LUS as anechoic, transonic images (Figure 1) in uncomplicated pleurisy.

The evidence of complicated pleural-effusion-associated hyperechoic flocculated fibrin strands (plankton sign) (Figure 3) was found on LUS in 6.7% of pneumonia patients; all of these patients were confirmed at CT, which was performed because of unfavorable evolution.

**Figure 4:**
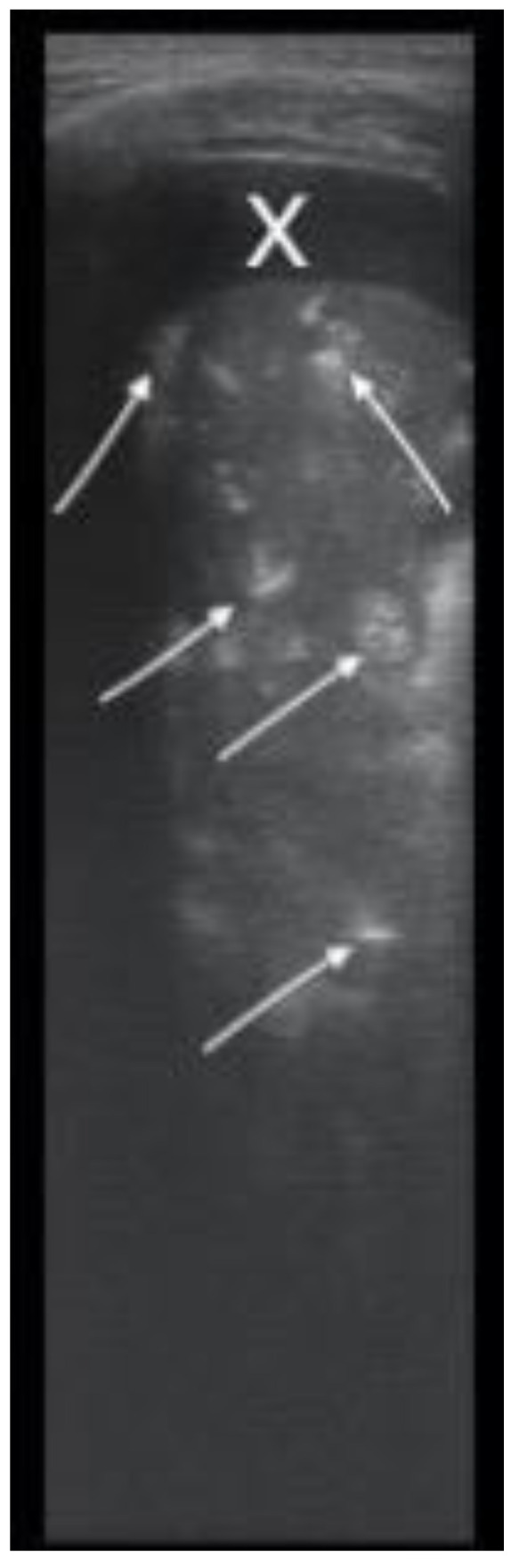
Consolidation with parenchymal structure and hyperechoic air (white arrows), showing the ventilation of the consolidation; adjacent pleural transonic effusion (X); 7–12 MHz, first day.

**Figure 5:**
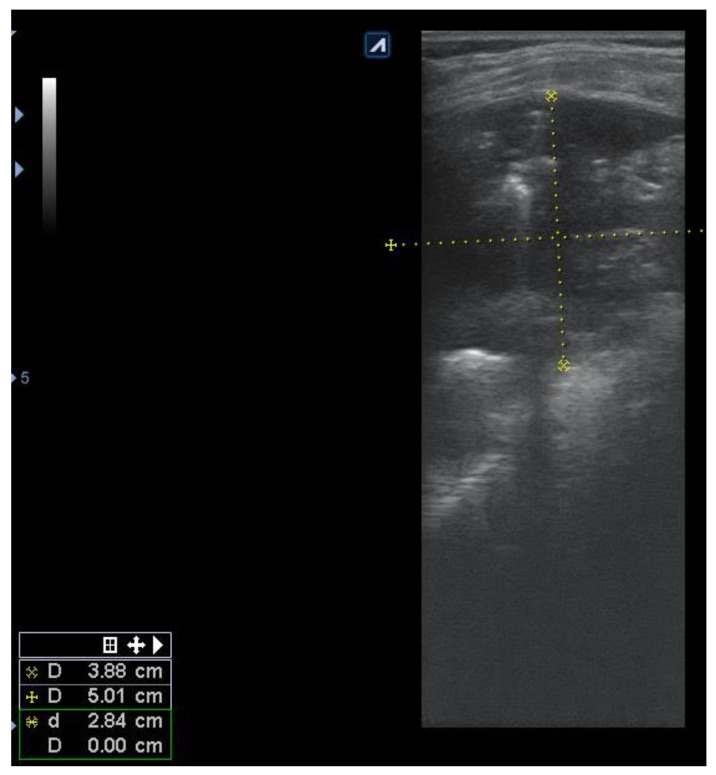
Persistence of consolidation (3.88/5.1 cm) with air bronchogram reabsorption of pleural effusion; 7–12 MHz, day 7.

Consolidations were easily detected by LUS as parenchymal liver-like structures, filled with hyperechoic images of the air that showed ventilation of the structures (Figure 4). The lesions were measured for an objective evaluation (Figure 5).

LUS was repeated for follow-up of the consolidations and verification of re-aeration by bronchogram; in the majority of cases (94.5%), a diminution of dimensions was registered, except in cases complicated by pleural abscess.

#### Pleural Effusions

**Figure 6:**
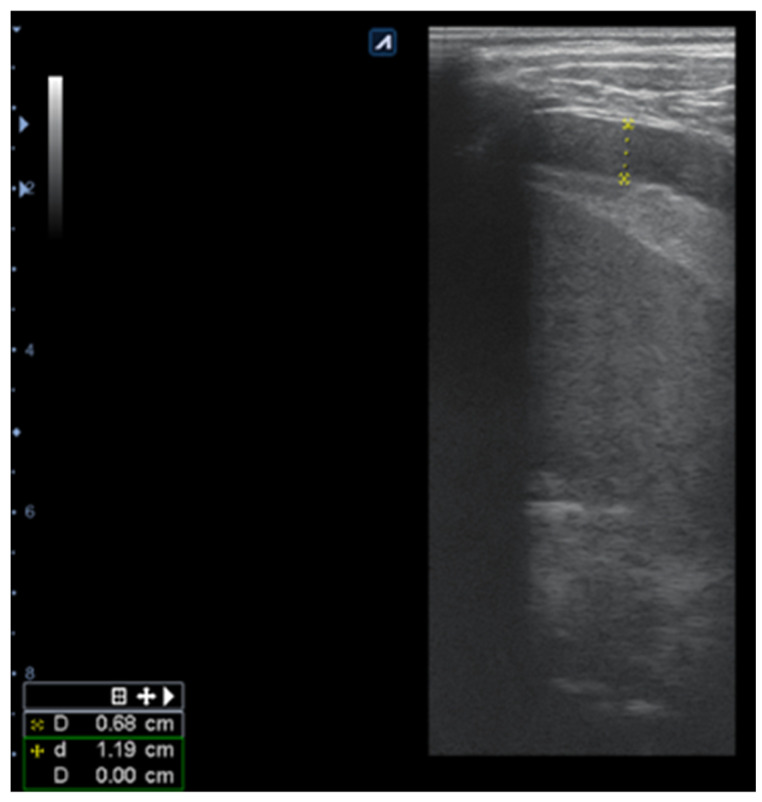
Pleural effusion (marked with X); interpleural distance of 0.68 cm, approximately 136 mL (distance × 20 = 136 mL).

**Figure 7:**
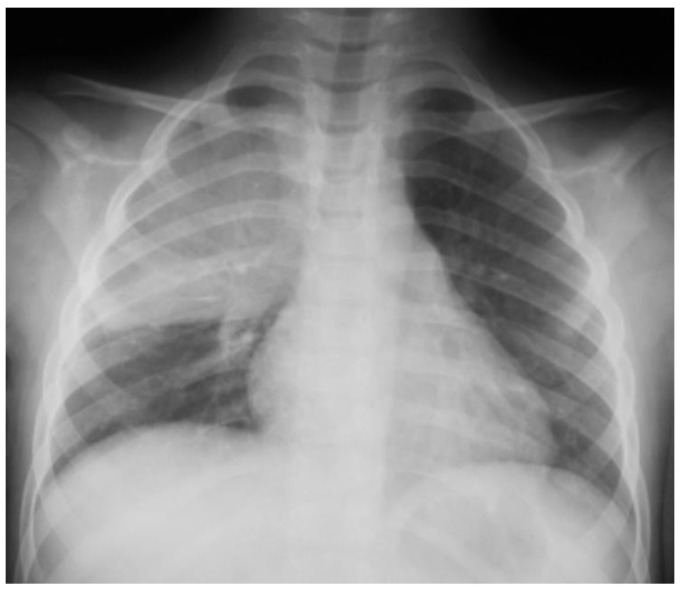
Opacity of medial right lobe, correspondent CXR.

A total of 14.8% of patients had pleural effusion (Figure 6), in four of whom complicated exudative pleurisy was found; pleural drainage was necessary in three of them. Lung ultrasound correctly detected the pleurisy (Figure 6), even if not clearly detected by CXR (Figure 7).

We calculated the volume of the pleural effusion using an equation in accordance with Sikora, 2012 [35] obtaining approximate 136 mL of pleural liquid.

The approximate volume of effusion could be calculated using an equation based on the interpleural distance—the measurement of distance between two sides of pleura; this calculation showed a very good approximation, verified only in patients (5.4%) who had evacuated pleural effusion.

LUS detected all 14.8% of patients with pleural effusion, compared to CXR, which diagnosed the presence of pleurisy only in 8.1% of patients, showing a lower sensitivity of 54.54% (CI 95% = 24.5622–81.2681%), compared to LUS, with maximum of 100% sensitivity for pleurisy diagnosis. The smaller pleural effusions were less easily detected by CXR, but more easily by LUS.

### 3.2. Agreement between LUS and CXR

#### 3.2.1. Consolidation Detection

Statistical estimation of the agreement between LUS and CXR in detection of the consolidation found an almost perfect agreement, with a Cohen’s kappa coefficient of K = 0.89 ± 0.04 SD and a statistically significant *p*-value of 0.000.

As we aimed to evaluate whether consolidations were detected by LUS and CXR, the almost unitary kappa coefficient sustained the agreement between the two methods, with a significant statistical significance regarding consolidation recognition.

#### 3.2.2. Pleural Effusion Detection Agreement

Furthermore, a substantial agreement of K = 0.67 ± 0.13 SD, *p* = 0.000 was found between the two methods—LUS and CXR—concerning the detection of pleural effusion in our group study. Although this agreement was substantial, the difference between the two methods was a result of the increased sensitivity of LUS in the diagnosis of pleurisy compared to CXR; nevertheless, both methods were reliable for the diagnosis of pleural effusion.

### 3.3. Sensibility and Sensitivity

The results of the study revealed a high sensitivity of 100% (CI 95% = 93.851–100%) for LUS for the detection of consolidated pneumonia, compared to CXR with a sensitivity of 90.5% (CI 95% = 80.9106–95.00%) (Table 3).

Both LUS and CXR had a good specificity of 100%, with a positive predictive value (PPV) of 100% (CI 95% = 93.851–100%) for LUS in detecting consolidations among children with clinical and biological signs of pneumonia. A negative predictive value (NPV) of 88.5% (CI 95% = 77.1721–94.00%) for chest X-ray was lower compared with LUS, which had a negative predictive value (NPV) of 100% (CI 95% = 91.7265–100%) for consolidation recognition

LUS was superior to CXR in sensitivity and negative predictive value, which emphasizes the fact that LUS detected consolidations more precisely than X-ray examination (Table 3) in our study.

CXR had a sensitivity of 90.5% (CI 95% = 80.9106–95.00%) and a specificity of 87% (CI 95% = 74.4835–94.00%) for pneumonia diagnosis. In our study, CXR described interstitial changes in seven patients, but without inflammatory markers; thus, the diagnosis of pneumonia was not established. The PPV of 90.5% (CI 95% = 80.91–95.00%) and NPV of 87% (CI 95% = 74.4835–94.00%) for CXR in detecting consolidations were lower compared to those of LUS.

**Figure 8:**
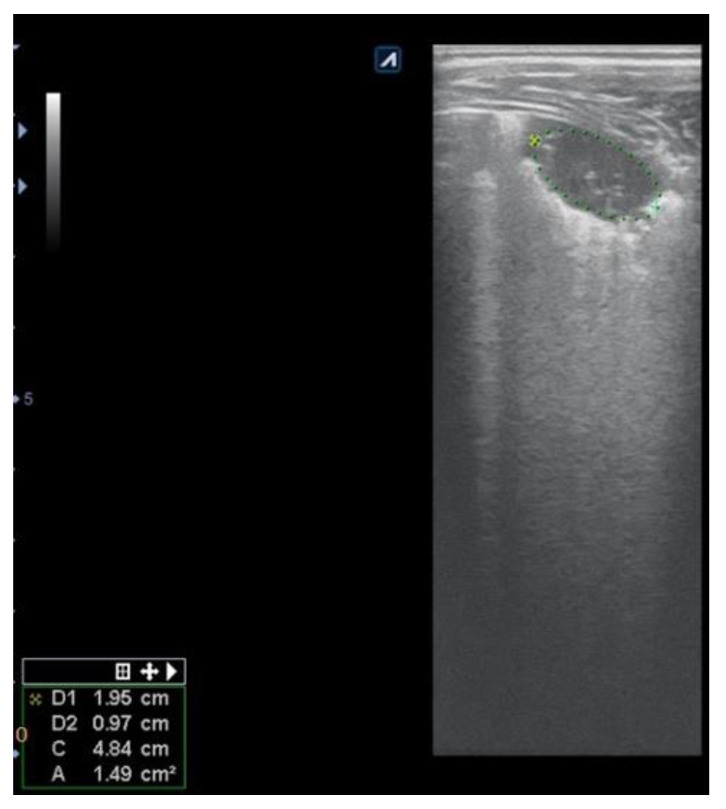
Subpleural consolidation, with air inside; dimensions: 1.96 cm/0.97 cm, 7–12 MHz linear transducer.

**Figure 9:**
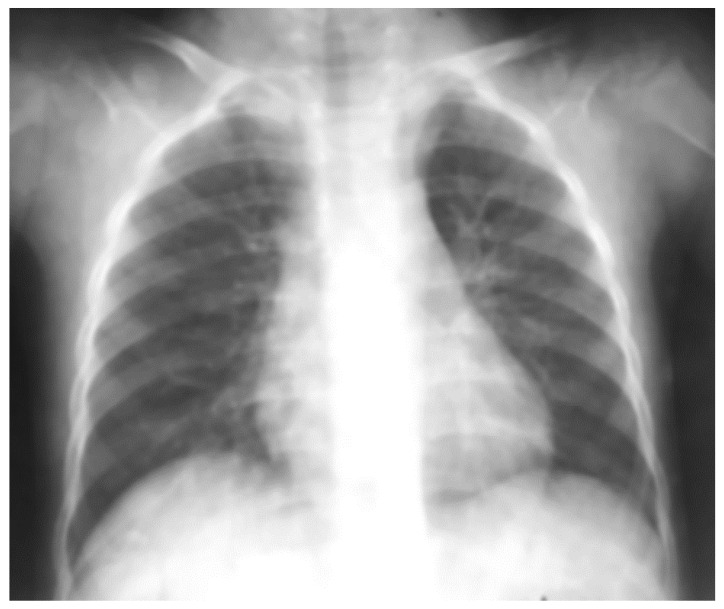
Correspondent CXR, no opacities.

Radio-occult pneumonia was diagnosed by LUS in 9.4% of patients (7 patients). For patients diagnosed with “echo pneumonia”, 90.5% of patients (67 patients) presented radiological characteristic images of pneumonia, while all “radiological-positive pneumonia” patients had confirmed LUS consolidations (Figure 8) prior to CXR (Figure 9).

Only four children with unfavorable evolution were subjected to a CT scan; in all patients the consolidations were detected by LUS and CXR, and confirmed by tomography.

## 4. Discussion

Pneumonia was diagnosed in our group with an increased prevalence among children below 6 years of age, comprising two-thirds of our study patients—an age group of children that are more susceptible to developing pneumonia [5,36]. The frequency of pneumonia was higher in our patients, as the study included a population of children with pneumonia partially immunized against pneumococcus; even though the vaccine has been available since 2017 in Romania, the coverage is low compared to other countries. Therefore, the low immunization rate is associated with increased pneumonia incidence, and also more severe forms of pneumonia.

Among the patients with pneumonia, a very slight male predominance of 51.35% was registered, consistent with other studies on another pediatric population [36] and adult pneumonia [37].

As we aimed to evaluate whether LUS is reliable for the definite diagnosis of pneumonia by the presence of consolidation and increase inflammatory markers, the results were encouraging.

For this study, we chose to specifically assess patients with consolidation, as consolidations are more likely to express parenchymal inflammatory injury compared to other artifacts. LUS patterns for normal lung aeration consist of the presence of A-lines with lung sliding, while with pneumonia progression, the loss of lung aeration, presence of B lines, subpleural consolidation, and loss of lung aeration occur, resulting from confluent pneumonia [38]. If an obstruction by mucous plugging or inflammation produces a subsequent atelectasis, the LUS image might be misinterpreted as consolidation, but differentiation between atelectasis and pneumonia can be made by the presence of several supplemental artifacts, such as the presence of air or fluid bronchogram, adjacent B lines (comet tails) and vascularization of the lesion [30].

As in unventilated areas, a remanence of the air inside the (unchanging with inflammation resolution and re-ventilation) lesion might be erroneously considered to be pneumonia; in our study, the follow-up of the lesion was very helpful for differentiation from atelectasis [38]. In our study, the follow-up of the lesions on the 3rd and 7th days of evolution made the confirmation of pneumonia easy, by the resolution of consolidation and extension of normal aeration. Lung ultrasound had an excellent sensitivity of 100%—better than chest X-ray (90.5%) for the diagnosis of consolidation in our study. These findings of the study are consistent with those found in the international literature, displaying the potential benefit of LUS compared to CXR in the diagnosis of community-acquired pneumonia in children [39,40,41].

The LUS was superior in terms of sensitivity for the detection of pleural effusion in our study, similar to the findings of other publications [10].

The results of our study thus show that the sensitivity of the lung ultrasound in detecting pneumonia is significantly higher in comparison with the chest X-ray, and has similar specificity. We concluded that the choice of the imaging method influences the obtaining of the diagnosis, while the chances of finding the diagnosis are similar with LUS.

An almost perfect agreement between LUS and CXR in the detection of consolidation was present, confirming the agreement between CXR and LUS regarding the existence of consolidated pneumonia, similarly to other reports [42], confirming that LUS can detect imagistic modification as well as CXR.

Even if CXR has a high negative predictive value for pneumonia in children [43], being factually considered the imaging standard for diagnosing pediatric pneumonia [44], in our study, CXR did not detect any consolidations among seven patients, significantly decreasing its sensitivity for pneumonia diagnosis, as found by studies comparing CXR with computed tomography [45]. We defined pneumonia by the presence of consolidation detected on LUS or CXR in patients with clinical signs and laboratory-increased inflammation markers; therefore, in patients with interstitial changes expressed at CXR, but without inflammatory markers, the diagnosis of pneumonia was not confirmed. One explanation might be the dimensions of the lesions—smaller lesions are not visible by CXR [46,47], as by LUS [23,40]. It was suggested that small lesions might be signs of either viral pneumonia [48] or atelectasis; therefore, subcentimetric subpleural consolidation was considered in our study only if dynamic bronchogram was present [28]. In several children, CXR revealed accentuated interstitial thickening that was not confirmed by LUS. The ultrasound-correspondent artifact for CXR peribronchial thickening would be the presence of pathological B lines [49], which were not found in these patients. The explanation might be the appearance of accentuated interstitium in CXR performed on children who are not always capable of executing the required inhalation during X-ray exposure.

As the presence of consolidation associated with biological inflammation suggests bacterial pneumonia, our results suggest that lung ultrasound in combination with biological study can reveal the pneumonia`s bacterial etiology, as in the study published by Berce et al. [42], who showed that LUS would be suitable for differentiating between etiologies [50]. In our study, we chose to include the presence of consolidation in pneumonia diagnosis, in order to be as accurate as possible with the pneumonia diagnosis; therefore, the presence of LUS consolidation overlapped with the presence of pneumonia among those 74 patients, giving LUS a maximum of 100% positive predictive value for community-acquired pneumonia. It should be noted that patients with consolidation detected before biochemical evaluation had positive inflammatory markers, suggesting—along with clinical picture and consolidation detection—a likely bacterial etiology. Nevertheless, it would have been very questionable to advance a theoretical supposed etiology of pneumonia for this study, as we did not have consistent bacteriological or serological proof of specific etiologies for these patients.

The results support the reliability of LUS, as stated in other studies [10,38,39,48,51], confirming that LUS is more sensitive than chest X-ray in the detection of pneumonia; therefore, ultrasound is an effective imaging test for the diagnosis of childhood pneumonia.

Both LUS and CXR had a good specificity of 100% for pneumonia diagnosis in our population selected by clinical signs and biological inflammation, showing that both imaging methods are precise for pneumonia diagnosis. The chest X-ray negative predictive value of NPV = 88.5% was lower compared with LUS, because in patients with a normal CXR, pneumonia—expressed by subpleural consolidations associated with inflammatory markers—was diagnosed by LUS in seven patients. LUS registered a negative predictive value of 100%; none of the 54 patients without detectable LUS lesions developed pneumonia.

A well-known pitfall of LUS would be that it does not detect the lesions that have not reached the pleural line [52], and lesions can hide near the scapula. Compared to adults, children have anatomical advantages that make them suitable for LUS, such as thinner chest walls, a lower ossification of thoracic bones, and lesser lung volumes [47].

Despite the rising evidence supporting the practice of lung ultrasound in several pathologies, a clear standardization is lacking, especially for pediatrics. The European Respiratory Society (ERS) elaborated a statement on thoracic ultrasound [15] for major zones of LUS practice and application, such as pneumonia. Nevertheless, even if LUS have a vast utility for pediatric pulmonary diseases, there is no standardization for the method, and an evidence-based approach would be necessary.

A few limitations exist for this study, which must be acknowledged: although significant for one year of study, the number of patients is still limited for wider applicability, and a larger, multicenter study would clearly provide more generalizable results. Another limitation is that a comparison between LUS and computed tomography—which is the gold standard examination for lung pathology—was not possible, as it was performed in just a few cases, owing to its potential for irradiation. A single radiologist interpreted the results; therefore, we also acknowledge this limitation. Finally, as we considered only pneumonia with consolidation detected, cases of pneumonia without consolidations were not included in the study, nor were artifacts such as B lines quantified. The selection was performed in this way in order to increase the sensitivity of pneumonia diagnosis associated with consolidation. Moreover, we concede that the lack of a confirmed pneumonia etiology in our study is another limitation, even if clinical positive inflammatory markers were part of the inclusion criteria. Regarding the subpleural consolidations, a wider critical approach would be important, as differentiation from atelectasis is very important, along with their capacity to suggest etiology. As stated previously, only consolidations with bronchogram were taken into consideration for this study. One single patient had a subcentimetric consolidation with bronchogram, and resolution of consolidation was noticed, along with re-ventilation, in the context of a typical clinical picture, with focalized crackles superjacent to LUS-detected lesions and positive inflammatory biomarkers.

Pulmonary ultrasonography is a non-irradiating, more easily accessible, and repeatable method of diagnosis that should play a leading role in the management of pediatric pneumonia.

According to our study, pulmonary ultrasound had a greater sensitivity than chest X-ray for assessing the diagnosis of consolidated pneumonia in hospitalized children, constituting an alternative imaging examination to chest X-ray for the diagnosis of pneumonia in children.

Consequently, the fastest, most reliable, and least harmful investigative method should be used as the first-line method, and LUS fulfils these criteria.

Pulmonary ultrasonography should be considered the imagistic method of choice for the screening of children with suspected pneumonia, even if a chest X-ray failed to detect consolidations.

## 5. Conclusions

Pneumonia is an important cause of mortality and morbidity in the pediatric population; therefore, a rapid recognition with as little radiation as possible would be preferable. In our study, lung ultrasound detected the presence of consolidations among children with pneumonia more accurately compared to CXR. Its sensitivity was also higher for the detection of pleural effusions, compared to X-ray. Thus, we suggest that LUS should be used as the first-line method of choice in the diagnosis of pediatric pneumonia.

## Figures and Tables

**Table 1:** Descriptive data and clinical signs of patients diagnosed with pneumonia compared to controls.

Characteristic *n* (%)	Pneumonia pts	Controls	Pneumonia pts	Controls	Pneumonia pts	Controls	Pneumonia pts	Controls
	Age	Age	Age	Age	Age	Age	Age	Age
	1–3 Years	1–3 Years	3–6 Years	3–6 Years	6–12 Years	6–12 Years	12+ Years	12+ Years
	*n* = 28 (37.8%)	*n* = 21 (38.9%)	*n* = 25 (33.8%)	*n* = 14 (25.9%)	*n* = 14 (18.9%)	*n* = 12 (22.2%)	*n* = 7 (9.5%)	*n* = 7 (13%)
Female	10 (35.7%)	9 (42.9%)	12 (48%)	8 (57.1%)	9 (64.3%)	9 (64.3%)	5 (71.4%)	3 (42.9%)
Male	18 (64.3%)	12 (57.1%)	13 (52%)	6 (42.9%)	5 (35.7%)	5 (35.7%)	2 (28.6%)	4 (57.1%)
Polypnea	27 (96.4%)	13 (54.2%)	23 (96.4%)	7 (50%)	11 (78.6%)	7 (58.3%)	4 (57.1%)	4 (57.1%)
Difficulty breathing	24 (85.7%)	11 (52.4%)	21 (84%)	6 (42.9%)	10 (71.4%)	7 (58.3%)	5 (71.4%)	5 (71.4%)
Chest retraction	24 (85.7%)	15 (71.4%)	20 (80%)	5 (35.7%)	4 (28.5%)	6 (50%)	2 (28.5%)	2 (28.6%)
Hypoxemia at admission (SpO_2_ < 95%)	17 (60.7%)	13 (62%)	15 (60%)	5 (35.7%)	8 (57.1%)	7 (58.3%)	3 (42.8%)	4 (57.1%)

pts: patients; SpO_2_: peripheral capillary oxygen saturation

**Table 2:** Percentages of severe pneumonia forms and mean values and SDs of patient’s SpO_2,_ leucocytes, CRP, and ESR, by age groups.

	Mean Value All Patients	Age	Age	Age	Age
		1–3 Years	3–6 Years	6–12 Years	12+ Years
		*n* = 28 (37.8%)	*n* = 25 (33.8%)	*n* = 14 (18.9%)	*n* = 7 (9.5%)
Severe pneumonia	NA	11 (39.28%)	12 (48%)	4 (28.5%)	1 (14.2%)
SpO_2_	94.4 ± 26.71	94.27 ± 2.34	94.3 ± 3.05	94.78 ± 2.72	95 ± 3.16
Leukocytes ×10^3^/mm^3^	18.04 ± 5.51	16.26 ± 4.7	18.88 ± 5.45	20.5 ± 6.67	17.2 ± 4.69
CRP mg%	127.04 ± 91.62	109.9 ± 97.1	146.24 ± 91.2	152.6 ± 82.8	75.6 ± 63.5
ESR mm/h	41.4 ± 22.21	36.8 ± 21.2	50.2 ± 24.9	39.5 ± 16.8	31.8 ± 18.3

SD: standard deviation; SpO_2_: peripheral capillary oxygen saturation; CRP: C-reactive protein; ESR: erythrocyte sedimentation rate.

**Table 3:** Sensitivity and specificity of LUS compared to CXR.

	Sensitivity	Specificity	PPV	NPV
	(CI 95%)	(CI 95%)	(CI 95%)	(CI 95%)
LUS consolidation	100%	100%	100%	100%
	(93.85–100)	(91.72–100)	(93.85–100)	(95.72–100)
CXR consolidation	90.5%	100%	100%	88.5%
	(80.91–95.00)	(91.72–100)	(93.85–100)	(77.17–94.00)
CXR with changes	90.5%	87%	90.5%	87%
	(80.91–95.00)	(74.48–94.00)	(80.91–95.00)	(74.48–94.00)

## Data Availability

Data is contained within the article or Supplementary Material.

## Supplementary Materials

The following are available online at https://www.mdpi.com/article/10.3390/children8080659/s1. Table S1: Descriptive patients database.
